# Haemoglobin(βK120C)–albumin trimer as an artificial O_2_ carrier with sufficient haemoglobin allostery†

**DOI:** 10.1039/d0cb00056f

**Published:** 2020-07-13

**Authors:** Yoshitsugu Morita, Asuka Saito, Jun Yamaguchi, Teruyuki Komatsu

**Affiliations:** Department of Applied Chemistry, Faculty of Science and Engineering, Chuo University, 1-13-27 Kasuga, Bunkyo-ku Tokyo 112-8551 Japan komatsu@kc.chuo-u.ac.jp

## Abstract

The allosteric O_2_ release of haemoglobin (Hb) allows for efficient O_2_ delivery from the lungs to the tissues. However, allostery is weakened in Hb-based O_2_ carriers because the chemical modifications of the Lys- and Cys-β93 residues prevent the quaternary transition of Hb. In this paper, we describe the synthesis and O_2_ binding properties of a recombinant Hb [rHb(βK120C)]–albumin heterotrimer that maintains sufficient Hb allostery. The rHb(βK120C) core, with two additional cysteine residues at the symmetrical positions on its protein surface, was expressed using yeast cells. The mutations did not influence either the O_2_ binding characteristics or the quaternary transition of Hb. Maleimide-activated human serum albumins (HSAs) were coupled with rHb(βK120C) at the two Cys-β120 positions, yielding the rHb(βK120C)–HSA_2_ trimer, in which the Cys-β93 residues were unreacted. Molecular dynamics simulation demonstrated that the HSA moiety does not interact with the amino acid residues around the haem pockets and the α_1_β_2_ surfaces of the rHb(βK120C) core, the alteration of which retards Hb allostery. Circular dichroism spectroscopy demonstrated that the quaternary transition between the relaxed (R) state and the tense (T) state of the Hb core occurred upon both the association and dissociation of O_2_. In phosphate-buffered saline solution (pH 7.4) at 37 °C, the rHb(βK120C)–HSA_2_ trimer exhibited a sigmoidal O_2_ equilibrium curve with the O_2_ affinity and cooperativity identical to those of native Hb (*p*_50_ = 12 Torr, *n* = 2.4). Moreover, we observed an equal Bohr effect and 2,3-diphosphoglycerate response in the rHb(βK120C)–HSA_2_ trimer compared with naked Hb.

## Abstract 2

Recombinant haemoglobin [rHb(βK120C)] was coupled with two human serum albumins (HSAs), yielding a rHb(βK120C)–HSA_2_ heterotrimer, which shows a sigmoidal O_2_ equilibrium curve and sufficient Hb allostery identical to those of native Hb.

## Graphical abstract

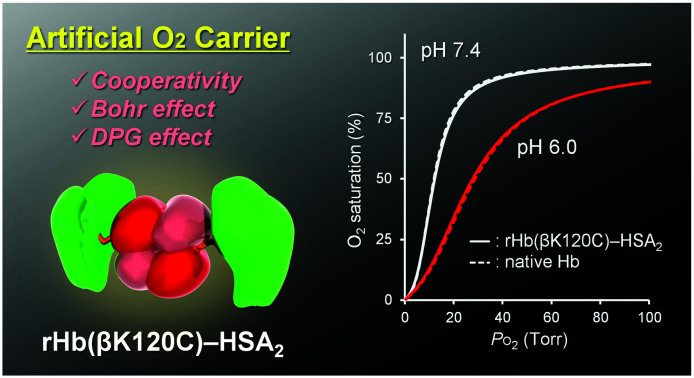

## Introduction

Allosteric regulation plays a crucial role in controlling the protein function in biological systems through the binding of a small-molecule effector that induces a conformational change. Haemoglobin (Hb) is a well-known allosteric protein encapsulated in red blood cells (RBCs),^1–5^ and its allostery has been extensively studied to understand its mechanism.^6–12^ O_2_ binding converts the quaternary structure of Hb from a deoxy tense (T) state with a low O_2_ affinity to a relaxed (R) state with a high O_2_ affinity.^1–4^ The homotropic allosteric effect, which is known as cooperativity, allows Hb to efficiently transport O_2_ from the lungs to the peripheral tissues. As a heterotropic allosteric effect, 2,3-diphospoglycerate (DPG) stabilizes the T-state conformation by creating salt bridges between the two β subunits to reduce O_2_ affinity.^13,14^ Furthermore, O_2_ affinity decreases with decreasing pH (the Bohr effect), shifting the equilibrium toward the T state.^11,15,16^

**Fig. 1:**
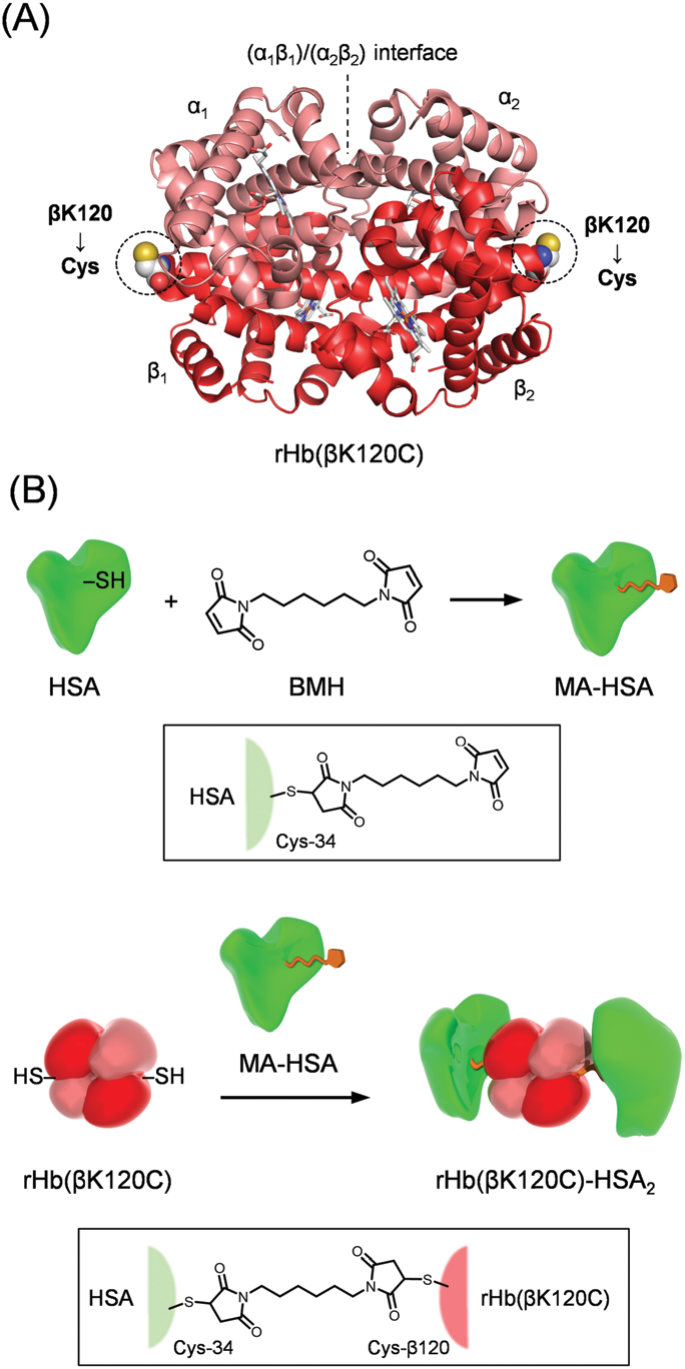
(A) Recombinant Hb variant [rHb(βK120C)] with Lys-β120 residues replaced by cysteines at the symmetrical positions. The mutation point was designed to avoid the haem pockets and α_1_β_2_ interfaces. (B) Synthetic routes of maleimide-activated HSA (MA-HSA) and the rHb(βK120C)–HSA_2_ trimer. The Cys-34 of the HSA was reacted with 1,6-bis(maleimido)hexane (BMH), resulting in MA-HSA. The two Cys-β120 residues at symmetrical positions were reacted with MA-HSA to yield the rHb(βK120C)–HSA_2_ trimer.

Artificial O_2_ carriers are currently required as RBC substitutes for transfusion therapy because of worldwide shortages in blood supply.^17,18^ However, the stroma-free Hb splits to an αβ dimer (α_2_β_2_ → 2αβ) in the bloodstream and is immediately expelled into urine. Moreover, Hb diffuses into the interstitial spaces between the endothelial cells and smooth muscle cells, causing hypertension by scavenging nitric oxide (NO; endothelial-derived vasodilator).^19,20^ Therefore, naked Hb is not available for use in transfusion therapy. Since the 1980s, several types of Hb-based O_2_ carriers have been developed, including cross-linked Hb,^21,22^ polymerized Hb,^23,24^ and PEGylated Hb.^25–27^ However, these chemical modifications have markedly reduced cooperativity, the Bohr effect, and the DPG effect,^18^ and a modified Hb that maintains the original allosteric effects of Hb has never been reported.^18^ We previously synthesized a protein cluster as an RBC substitute consisting of an Hb molecule wrapped covalently in human serum albumins (HSAs), called an Hb–HSA_*m*_ cluster (*m* = 2–4, average *m* = 3.0 ± 0.2).^28,29^ This protein cluster shows a long circulation lifetime in the bloodstream and a superior biocompatibility.^30,31^ The quaternary structural analysis of the Hb–HSA_*m*_ cluster revealed that the chemical modification of the Lys and Cys-β93 residues of Hb by *N*-succinimidyl 3-maleimidopropionate as a cross-linker prevents the R–T quaternary transition and maintains the Hb core close to the R state.^32^ Thus, the Hb–HSA_*m*_ cluster demonstrated a low cooperativity, a low Bohr effect, and a low DPG effect. If the HSA shells are linked at appropriate positions on the Hb surface so that they do not affect the R–T quaternary motion, the resulting Hb–HSA_*m*_ conjugate maintains the original O_2_ binding properties of the native Hb. The genetically introduced cysteine could act as a cross-linking point for maleimide-activated HSA (MA-HSA). We assume that the mutation of Hb residues, excluding the mutation of (i) the α_1_β_2_ interfaces, including the N- and C-terminals (which play important roles in the R–T quaternary transition), and (ii) the haem pockets (which directly affect O_2_ affinity), would allow the resulting Hb variant to maintain its original O_2_ binding capability. Based on this hypothesis, the Lys-β120 residues were chosen as the symmetrical mutation points (Fig. 1A). In this study, we describe a novel recombinant Hb variant, rHb(βK120C), in which Lys-β120 is replaced with Cys at symmetrical positions on the Hb surface, and we also present the rHb(βK120C)–HSA_2_ heterotrimer, in which MA-HSAs are linked at the Cys-β120 positions (Fig. 1B). Ultraviolet-Visible (UV-vis) and circular dichroism (CD) spectroscopies revealed that the quaternary structure of the rHb(βK120C)–HSA_2_ trimer is reversibly convertible between the R and T states. We also observed identical O_2_ affinity and allosteric effects in the rHb(βK120C)–HSA_2_ trimer compared with naked Hb.

## Results and discussion

### Expression and physicochemical properties of rHb(βK120C)

**Fig. 2:**
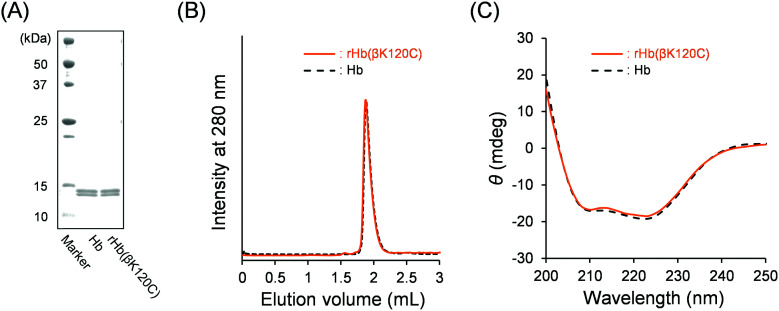
(A) SDS–PAGE results of native Hb and rHb(βK120C). (B) SEC profiles of rHb(βK120C) and native Hb. (C) CD spectra of the rHb(βK120C) and native Hb in PBS at 25 °C ([protein] = 0.2 μM).

We first expressed rHb(βK120C) using *Pichia* yeast (*Pichia pastoris* GS115) according to our previous reports.^33,34^ The expressed rHb(βK120C) was purified using cation- and anion-exchange chromatographies (CEC and AEC). The sodium dodecyl sulfate–polyacrylamide gel electrophoresis (SDS–PAGE) analysis of rHb(βK120C) depicted two clear bands corresponding to the α- and β-subunits (Fig. 2A). The α- and β-chain bands demonstrated near-identical mobility to those of native Hb. The matrix-assisted laser desorption/ionization-time of flight (MALDI-TOF) mass spectrum analysis of rHb(βK120C) exhibited molecular-related ion peaks at 15 122 and 15 841 Da. These were almost identical to the simulated masses of the amino acid sequences (15 127 Da for the α-chain and 15 842 Da for the β-chain), indicating that Lys-β120 was replaced with cysteine. The size-exclusion chromatography (SEC) chromatogram of rHb(βK120C) exhibited a peak with the same elution volume as that of native Hb, indicating that the tetramer (α_1_α_2_β_1_β_2_) was formed in the rHb(βK120C) (Fig. 2B; elution volume = 1.9 mL). Fortunately, the oligomeric rHb(βK120C) species created *via* the disulfide bonds of Cys-β120 was not observed in the SEC chromatogram. The fact that rHb(βK120C) and native Hb had identical CD spectra (*λ* = 200–250 nm) indicates that their secondary structures were equivalent (Fig. 2C). The isoelectric point (pI) value of rHb(βK120C) was determined to be 6.8, which was slightly lower than that of native Hb (pI = 6.9) because the mutation (Lys-β120 → Cys) had negatively shifted the net surface charge (Fig. S1, ESI†). The results of the sulfhydryl group assay using 4,4′-dithiodipyridine (4,4′-DTP)^35^ revealed the number of reactive sulfhydryl groups in rHb(βK120C) and native Hb as 4.0 and 2.0, respectively. This result strongly indicates that the two Lys-β120 residues of the β_1_ and β_2_ chains were replaced by Cys and that the introduced Cys-β120 residues were reactive with 4,4′-DTP, as they are located at the molecular surface of rHb(βK120C).

### Preparation of the rHb(βK120C)–HSA_2_ trimer

To prepare MA-HSA, we used 1,6-bis(maleimido)hexane (BMH) as the cross-linking agent. One of the two maleimide groups in BMH was reacted with the free sulfhydryl group of the Cys-34 in HSA (which is the only reduced-form of cysteine in HSA), yielding MA-HSA. The formation of the HSA dimer was not observed because of electrostatic repulsion between the negatively charged HSA surfaces present under our experimental conditions. The sulfhydryl group assay using 4,4′-DTP revealed the complete disappearance of the reduced form.

**Fig. 3:**
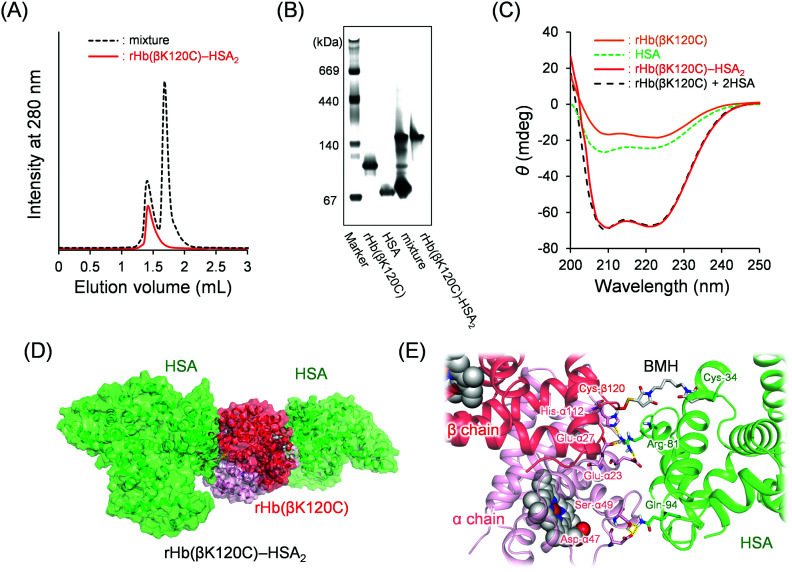
(A) SEC profiles of the resultant reaction mixture [rHb(βK120C) + MA-HSA] and the purified rHb(βK120C)–HSA_2_ trimer. (B) Native PAGE patterns of rHb(βK120C), HSA, the reaction mixture, and the purified rHb(βK120C)–HSA_2_ trimer. (C) CD spectra of rHb(βK120C), HSA, rHb(βK120C), and rHb(βK120C)–HSA in PBS at 25 °C ([protein] = 0.2 μM). (D and E) The energy-minimized structure of the rHb(βK120C)–HSA_2_ trimer obtained by a molecular dynamics (MD) calculation using the Desmond software.

Capping of the Cys-β93 residues of native Hb with maleimide derivatives causes a decline in O_2_ binding cooperativity, the Bohr effect, and the influence of the allosteric effectors. The Cys-β93 residues of native Hb were able to react with maleimide derivatives, such as *N*-ethyl maleimide (NEM) and *N*-succinimidyl 3-maleimidopropionate.^32,36^ We found that the Cys-β93 residues had not reacted with MA-HSA because of the steric hindrance between the native Hb and MA-HSA. In contrast, genetically introduced Cys-β120 located at the molecular surface of rHb(βK120C) had reacted with MA-HSA. The SEC chromatogram of the reaction mixture [rHb(βK120C) + MA-HSA] revealed a new peak in the high-molecular-weight region (Fig. 3A; elution volume = 1.4 mL), and native PAGE analysis also revealed a new band above that of rHb(βK120C) (Fig. 3B). The high-molecular-weight component was purified using AEC followed by gel filtration chromatography (GFC). The ion peak of the β-chain at 15.8 kDa in the MALDI-TOF MS disappeared after the reaction of rHb(βK120C) with MA-HSA. A new peak then appeared at 82.5 kDa, a value almost identical to the simulated mass of a β-chain + MA-HSA (82.3 kDa), indicating that the HSA moiety was covalently linked to the β-chain of rHb(βK120C). Based on the [total protein]/[Hb unit] assays, the average HSA/Hb ratio of the product was determined to be 2.0. The heme loss was not observed during the preparation. We denoted this hybrid protein as rHb(βK120C)–HSA_2_ (*M*_w_: 198 kDa). The CD spectrum of the rHb(βK120C)–HSA_2_ trimer coincided perfectly with the sum of the rHb(βK120C) spectrum and a two-times-enlarged HSA spectrum (Fig. 3C). This result demonstrates that the secondary structures of the individual proteins were unaltered by the protein coupling. The dynamic light scattering measurements revealed that the hydrodynamic diameter of the rHb(βK120C)–HSA_2_ trimer (12.4 ± 2.8 nm, P.I. = 0.010) was significantly larger than that of rHb(βK120C) (6.9 nm ± 1.9 nm, P.I. = 0.004). The pI value of the trimer (pI = 5.4) was close to that of HSA (pI = 4.9), which further indicates the coupling of rHb(βK120C) (pI = 6.7) with HSAs (Fig. S1, ESI†). The net negative surface charge and the large molecular size could allow the rHb(βK120C)–HSA_2_ trimer to circulate in the bloodstream for long periods of time and prevent the undesirable vasopressor response through NO depletion.^19,20^ Furthermore, the sulfhydryl group assay using 4,4′-DTP^35^ demonstrated that the number of reactive sulfhydryl groups in the rHb(βK120C)–HSA_2_ trimer was 1.8, indicating that the Hb core maintained the free Cys-β93 residues that are important for O_2_ binding ability, as mentioned above.^36^ We could not observe the dissociation of the α_2_β_2_ tetramer into two αβ dimers during the synthesis, purification, and measurements of rHb(βK120C)–HSA_2_, implying that the α_2_β_2_ tetramer was sufficiently stable the same as native Hb under our experimental conditions.

An MD simulation was used to predict the interactions between the rHb(βK120C) and HSA moieties. The Cys-β_1_120 and Cys-β_2_120 residues exist at symmetrical positions of the core Hb; therefore, a sufficient distance is maintained between the two HSA parts in the rHb(βK120C)–HSA_2_ trimer. We performed MD simulation using a half model [αβ(K120C)–HSA] constructed using the crystal structures of native Hb (PDB ID: 2DN1)^37^ and HSA (PDB ID: 1AO6)^38^ (Fig. S2, ESI†). The main chains (Cα and amide atoms) of the αβ(K120C) moiety were frozen for the duration of a 50 ns simulation. The root-mean-square deviation (RSMD) and the distances of the salt bridges and hydrogen bonds between the αβ(K120C) and HSA residues reached a plateau within 25 ns (Fig. S3, ESI†). The MD simulation structure was represented in the rHb(βK120C)–HSA_2_ form which was prepared by overlapping the two αβ(K120C) moieties of the models with the crystal structure of native Hb (α_1_β_1_α_2_β_2_; Fig. 3D and Fig. S2B, ESI†). The distance between the sulfide atoms of Cys-β120 [rHb(βK120C)] and Cys-34 (HSA) was measured to be 13.8 Å, meaning that the BMH was long enough to cross-link the Cys residues (Fig. 3E). We found that the following salt bridges and hydrogen bonding interactions between rHb(βK120C) and HSA helped stabilize the conformation of the rHb(βK120C)–HSA_2_ trimer: (i) Glu-α23, Glu-α27, and His-α112 of rHb(βK120C)—Arg-81 of HSA and (ii) Ser-α49 and Asp-α47 of rHb(βK120C)—Gln-94 of HSA. The HSA moiety did not interact with the amino acid residues around the haem pockets and the α_1_β_2_ surfaces of the rHb(βK120C) core.

### Quaternary structure analysis of the Hb core

**Fig. 4:**
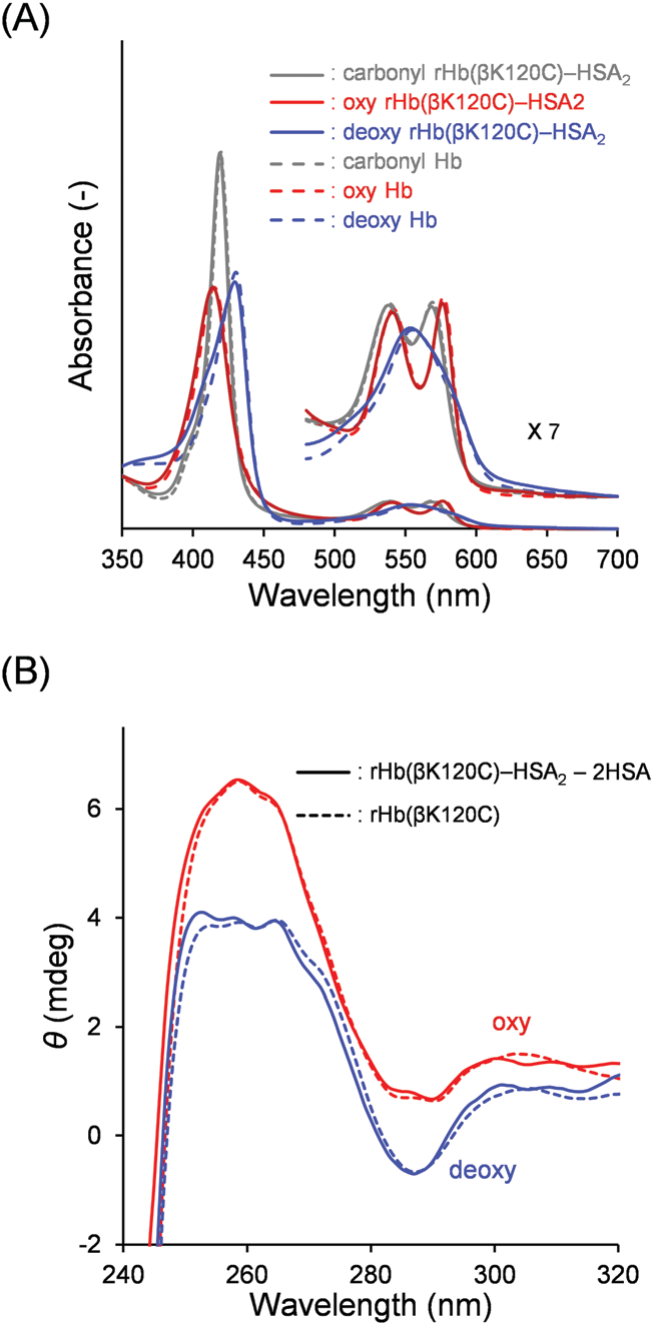
(A) UV-vis absorption spectra of the carbonyl (gray), oxy (red), and deoxy (blue) rHb(βK120C)–HSA_2_ trimer (solid line) and native Hb (dotted line). (B) CD spectra (240–320 nm) of the oxy (red) and deoxy (blue) Hb components of the rHb(βK120C)–HSA_2_ trimer [rHb(βK120C)–HSA_2_ − twofold HSA] (solid line) and rHb(βK120C) (dotted line). The measurements were performed in PBS solution (pH 7.4) at 25 °C ([protein] = 3 μM).

We investigated the quaternary structure of the Hb core using UV-vis absorption and CD spectroscopies. The UV-vis spectral patterns and the absorbances of the oxy, deoxy, and carbonyl rHb(βK120C) as well as the rHb(βK120C)–HSA_2_ trimer were indistinguishable from those of native Hb in PBS solution (pH 7.4; Fig. 4A and Table S1, ESI†). Generally, the chemical modification of the Lys and Cys-β93 residues of Hb decreases the Soret band absorption of the deoxy form, because the deoxy Hb remains near the R-state quaternary structure.^32^ In contrast, the deoxy rHb(βK120C) and the rHb(βK120C)–HSA_2_ trimer revealed the same Soret band absorption as that of deoxy native Hb, indicating that the identical T-state quaternary structures were formed. CD spectroscopy was also used to analyse the quaternary structure of the Hb core in the rHb(βK120C)–HSA_2_ trimer. Generally, deoxy Hb (T state) exhibits a negative CD band at 287 nm, which is known as the T-state marker.^39–41^ At the same time, this band is not usually observed in the R-state quaternary structure of oxy Hb. The CD spectral patterns and intensities of oxy and deoxy rHb(βK120C) both resembled those of native Hb, implying that the oxy and deoxy forms of these proteins are comparable (Fig. 4B).^39^ CD also revealed similar spectral changes in the rHb(βK120C)–HSA_2_ trimer upon deoxygenation. CD spectra for the oxy and deoxy Hb cores in the rHb(βK120C)–HSA_2_ trimer were obtained by subtracting the two-times-enlarged HSA spectrum from the corresponding rHb(βK120C)–HSA_2_ trimer spectra (Fig. 4B). The CD spectra of the oxy and deoxy Hb cores coincided with those of rHb(βK120C). These spectral analyses revealed that the mutation Lys-β120 → Cys and coupling of HSA at the Cys-β120 positions did not influence the quaternary structural transition between the R and T states.

### O_2_ binding affinity and cooperativity

**Fig. 5:**
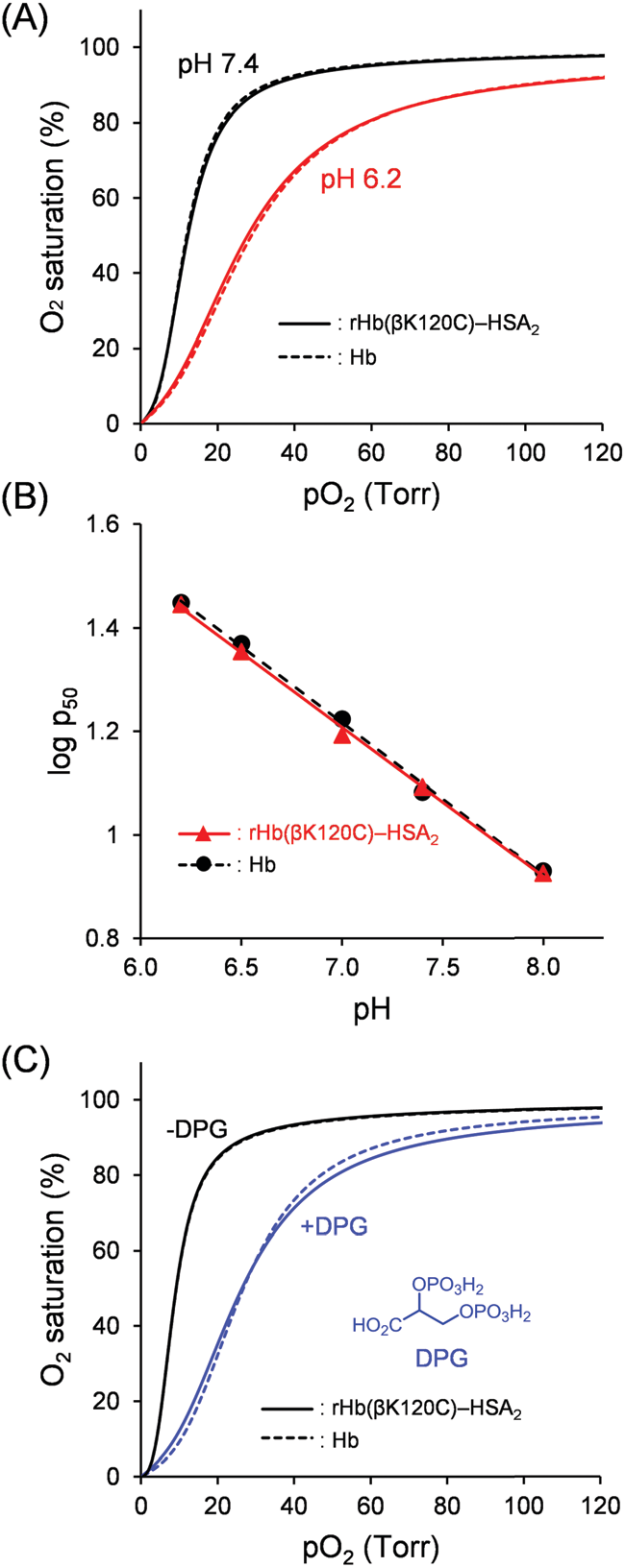
OECs and Bohr plots of Hb (dotted line) and the rHb(βK120C)–HSA_2_ trimer (solid line) at 37 °C ([protein] = 5 μM). (A) OECs in PBS solutions at pH 7.4 (black) and pH 6.2 (red). (B) The Bohr plots of Hb (black circle) and the rHb(βK120C)–HSA_2_ trimer (red triangle) in PBS solutions (pH 6.2, 6.5, 7.0, 7.4, and 8.0). (C) OECs in 50 mM Tris–HCl buffer solution with 1 mM DPG (blue) and without DPG (black).

Hb binds with O_2_ in the lungs, where there is a high O_2_ partial pressure (*p*O_2_), and releases O_2_ into the peripheral tissues, where there is a low *p*O_2_. To evaluate the O_2_ binding properties, O_2_ affinity (*p*_50_; *p*O_2_ where Hb is half-saturated with O_2_) and the Hill coefficient (*n*; degree of cooperativity) of Hb were determined from the O_2_ equilibrium curve (OEC). As shown in Fig. 5A, a sigmoidal OEC was observed for native Hb in PBS solution (pH 7.4) at 37 °C (*p*_50_ = 12 Torr; *n* = 2.4). Upon the release of O_2_, the quaternary structure of Hb converts from the R state with a high O_2_ affinity to the T state with a low O_2_ affinity. The cooperative O_2_ release allows Hb to efficiently deliver O_2_ from the lungs to the tissues. Nevertheless, chemically modified Hbs have demonstrated a low cooperativity because the quaternary transition of the Hb core is prevented.^32^ Such modified Hbs include PEG-Hb (71% reduction),^18,42^ polymerized Hb (41% reduction),^18,42^ and Hb–HSA_*m*_ clusters (71% reduction).^29,42^ In contrast, the OECs of rHb(βK120C) and the rHb(βK120C)–HSA_2_ trimer were found to have a sigmoidal shape completely consistent with that of native Hb (Fig. 5A). The O_2_ binding parameters of rHb(βK120C) (*p*_50_ = 12 Torr, *n* = 2.3) and the rHb(βK120C)–HSA_2_ trimer (*p*_50_ = 12 Torr, *n* = 2.4) were also equivalent to those of native Hb (Table 1). Both rHb(βK120C) and the rHb(βK120C)–HSA_2_ trimer were able to maintain identical O_2_ affinity and cooperativity because the quaternary structure of the Hb units was interconvertible between the R and T states upon O_2_ association and dissociation, as in native Hb. The capping of Cys-β93 with NEM in native Hb (NEM-Hb) reduced the *p*_50_ and *n* values (*p*_50_ = 8 Torr, *n* = 1.9). The NEM-rHb(βK120C)–HSA_2_ trimer demonstrated O_2_ binding parameters similar to those of NEM-Hb, clearly indicating that the Cys-β93 residues in the rHb(βK120C)–HSA_2_ trimer were in a reduced form. This result is supported by the sulfhydryl group assay mentioned above. We concluded that the selective coupling of MA-HSA occurred at the two Cys-β120 positions of the rHb(βK120C), whereas it did not take place at the Cys-β93 residues because of steric hindrance. On the basis of these findings, we also concluded that the Lys-β120 → Cys mutation and the selective conjugation of MA-HSA at the Cys-β120 positions were impervious to O_2_ affinity and cooperativity. To the best of our knowledge, this is the first example of a chemically modified Hb showing an identical O_2_ affinity and cooperativity to those of native Hb.^18^

**Table 1:** O_2_ binding parameters and Bohr coefficients of haemoproteins in PBS solution at 37 °C

Haemoprotein	pH 7.4		pH 6.2		Bohr coefficient^a^
	*p*_50_ (Torr)	*n* (—)	*p*_50_ (Torr)	*n* (—)	
Hb	12	2.4	28	1.9	0.29
rHb(βK120C)	12	2.3	29	1.9	0.29
rHb(βK120C)–HSA_2_	12	2.4	28	1.9	0.29

a The Bohr coefficients were derived from the slopes obtained from plotting −Δlog *p*_50_/ΔpH between pH 6.2 and 8.0 (Fig. 5B).

The autoxidation rate constant (*k*_ox_) of the oxygenated Hb core in the rHb(βK120C)–HSA_2_ trimer was measured in PBS solution (pH 7.4) at 37 °C. The *k*_ox_ value of the rHb(βK120C)–HSA_2_ trimer was ascertained as 0.027 h^−1^, which was almost similar to the data of native Hb (0.020 h^−1^). The oxy form of the internal Hb maintains good stability even after being covered with HSAs.

### Bohr effect on rHb(βK120C)–HSA_2_ trimer

A high metabolic activity leads to an increase in the concentration of CO_2_ and a decrease in the pH value within the tissues. Under low-pH conditions, the protonation of Hb occurs and the T-state quaternary structure is stabilized, reducing O_2_ affinity (the Bohr effect). Thus, the OEC of native Hb was right-shifted to a *p*_50_ value of 28 Torr at pH 6.2 compared with *p*_50_ = 12 Torr at pH 7.4 (Table 1 and Fig. 5A). The Bohr effect allows for Hb high O_2_-transport efficiency to the tissues. However, chemical modifications have tended to notably reduce the Bohr effect, such as in PEG-Hb (87% reduction)^18^ and polymerized Hb (75% reduction).^18^ We observed that the O_2_ affinities of rHb(βK120C) and the rHb(βK120C)–HSA_2_ trimer were reduced under lower pH conditions, similar to native Hb (*p*_50_: 12 → 29 Torr (pH 7.4 → 6.2)). The *p*_50_ and *n* values of both rHb(βK120C) and the rHb(βK120C)–HSA_2_ trimer were nearly identical to those of native Hb at pH 6.2 (Table 1). The Bohr coefficients were derived from the slopes obtained from plotting −Δlog *p*_50_/ΔpH between pH 6.2 and 8.0 (Fig. 5B and Table S2, ESI†). The Bohr coefficients of both rHb(βK120C) and the rHb(βK120C)–HSA_2_ trimer were identical to those of native Hb (Table 1). The value measured for native Hb, however, was somewhat weaker than the result reported by Ho *et al.*^43^ This difference is probably attributable to several factors, such as the difference in temperature (37 °C *vs.* 29 °C) and the difference in solvents (PBS with low phosphate and high NaCl concentrations *vs.* 0.1 M sodium phosphate buffer (PB)). Altogether, we conclude that neither the Lys-β120 → Cys mutation nor the HSA conjugation affected the Bohr effect.

### Effects of DPG on O_2_ binding affinity

Allosteric effectors bind to proteins, altering their activity. The most important allosteric regulator of Hb in RBCs is DPG, which reduces the O_2_ binding affinity (the DPG effect). An X-ray crystallography analysis has previously revealed that a single DPG molecule binds to the cleft of Hb β chains, known as the DPG binding site.^13,14^ The anionic groups of DPG form salt bridges with the cationic groups of the β-chains, including the His-β2, Lys-β82, and His-β143 residues, stabilizing the T-state conformation and reducing O_2_ affinity. The T → R conformational change induced by oxygenation leads to the closure of the binding site and weakens the DPG binding affinity to oxy Hb. This DPG response allows Hb to efficiently deliver O_2_ from the lungs to the tissues. We measured the OEC of native Hb in PBS (pH 7.4) solution containing 1 mM DPG at 37 °C, but a reduction in O_2_ affinity was not observed because of the fact that phosphate anions in the PBS solution interrupt the binding of DPG to Hb. Thus, we used 0.1 M Tris–HCl buffer for DPG response experiments. Indeed, native Hb in Tris–HCl buffer demonstrated a higher O_2_ affinity (*p*_50_ = 9 Torr) than in PBS solution because of the exclusion of the Hb–phosphate anion interactions (Table 2). The O_2_ affinity of the native Hb was also reduced upon the addition of DPG to Tris–HCl buffer. Interestingly, rHb(βK120C) (*p*_50_ = 26 Torr) and the rHb(βK120C)–HSA_2_ trimer (*p*_50_ = 26 Torr) demonstrated identical DPG responses to native Hb (*p*_50_ = 27 Torr; Fig. 5C). These results indicate that the rHb(βK120C)–HSA_2_ trimer maintained a DPG response similar to that of native Hb and that its O_2_ affinity is controllable *via* the allosteric effector.

**Table 2:** Effect of DPG on hemoproteins in 50 mM Tris–HCl buffer solution (pH 7.4) at 37 °C containing 1 mM DPG

Hemoprotein	Without DPG		With DPG	
	*p*_50_ (Torr)	*n* (—)	*p*_50_ (Torr)	*n* (—)
Hb	9	2.3	27	2.4
rHb(βK120C)	9	2.3	26	2.1
rHb(βK120C)–HSA_2_	9	2.3	26	2.1

## Conclusions

A novel Hb variant (Lys-β120 → Cys) was designed to prepare the rHb(βK120C)–HSA_2_ trimer as an artificial O_2_ carrier. The mutation did not affect the O_2_ binding properties of Hb. Two MA-HSA molecules were selectively linked to Cys-β120 in rHb(βK120C), resulting in the rHb(βK120C)–HSA_2_ trimer in which Cys-β93 remained free because of steric repulsion between the rHb(βK120C) core and the MA-HSA shell. The CD spectra revealed that the R–T quaternary motion of the Hb core in the rHb(βK120C)–HSA_2_ trimer occurred upon O_2_ association and dissociation. Thus, the rHb(βK120C)–HSA_2_ trimer demonstrated sufficient allosteric O_2_ binding properties with respect to cooperativity, the Bohr effect, and the DPG effect. The rHb(βK120C)–HSA_2_ trimer is a unique artificial O_2_ carrier with sufficient allostery to be an RBC substitute.

## Materials and methods

### Materials and apparatus

1,6-Bis(maleimido)hexane (BMH) was purchased from Tokyo Chemical Industry Co., Ltd. Human serum albumin (HSA, albumin 25%, Benesis) was purchased from the Japan Blood Products Organization. The other special-grade chemicals were used without additional purification unless otherwise noted. Water was deionized (18.2 MΩ cm) using two water purification systems (Elix UV and Milli Q Reference; Millipore Corp.). The SDS–PAGE and native PAGE analyses were performed using a 15% or 5–12% poly(acrylamide) precast gel (SuperSep Ace 15%; SuperSep Ace 5–12%; Fujifilm Wako Pure Chemical Corp.). Isoelectric focusing (IEF) was performed using a pH 3–10 IEF protein gel (Novex; Thermo Fischer Scientific Inc.). The UV-vis absorption spectra were obtained using a UV-Visible spectrophotometer (8543; Agilent Technologies Inc. or V-650; Jasco Corp.). CD spectra were recorded using a CD spectrometer (J-820; Jasco Corp.) at 25 °C.

### Expression and preparation of rHb(βK120C)

The expression plasmid for the rHb(βK120C) variant [pHIL-D2-rHb(βK120C)] was constructed according to the standard protocol of the QuickChange XL Site-Directed Mutagenesis Kit (Agilent Technologies Inc.) using a pHIL-D2-rHb(α- and β-chains) vector^34^ and an oligonucleotide primer set (forward [5′-CTCGCGCATCACTTCGGCT̲G̲T̲GAGTTTACACCGCCAGTTC-3′] and reverse [5′-GAACTGGCGGTGTAAACTCA̲C̲A̲GCCGAAGTGATGCGCGAG-3′]). The obtained pHIL-D2-rHb(βK120C) vector was linearized with *Sal*I and used to transform the GS115-strain *Pichia pastoris* (Thermo Fisher Scientific K.K.) *via* electroporation.

Transformed clone cells were grown in a buffered mineral glycerol complex (BMGY) medium (4 L total) in a shaking incubator (Bio-Shaker G·BR-200; Taitec Corp.; 200 rpm, 30 °C) and subsequently in a buffered mineral methanol complex (BMMY) medium (1.6 L total) containing 0.3 mM hemin for 5 days. During cultivation, 100% methanol at 1.5% of the medium volume was added every 24 h. The cells were harvested by centrifugation at 3000*g* for 10 min. The obtained cells were then washed with water (300 mL × 2) and resuspended in 100 mL of sodium PB solution (10 mM, pH 6.0) containing 1 mM phenylmethanesulfonyl fluoride. After the addition of glass beads (150 mL, *d* = 0.5 mm), the cells were lysed using a BeadBeater (Biospec Products, Inc.) with four cycles of disruption (2 min) and incubated by cooling on ice (2 min). After centrifugation at 12 000*g* for 1 h, the supernatant was equilibrated with CO atmosphere. The solution was loaded onto a cation exchange chromatography column (SP Sepharose Fast Flow, GE Healthcare UK Ltd), which was equilibrated in 10 mM PB (pH 6.0). After washing with the same buffer solution, the target protein was eluted using 20 mM Tris–HCl buffer solution (pH 8.0). Next, the resulting protein solution was subjected to AEC using a Q Sepharose Fast Flow column (GE Healthcare UK Ltd) with 20 mM Tris–HCl (pH 8.0) as the running buffer. After washing with 20 mM Tris–HCl (pH 8.0), rHb(βK120C) was eluted using PBS solution (pH 7.4). The purity was checked using SDS–PAGE analysis and SEC on a high-performance liquid chromatography (HPLC) system (AKTA purify; GE Healthcare UK Ltd). The system was equipped with an SEC column (Superdex 200 Increase, 5/150 GL; GE Healthcare UK Ltd), and PBS solution (pH 7.4) was used as the mobile phase. The concentration of rHb(βK120C) was measured using a protein assay kit (Pierce 660 nm; Thermo Fisher Scientific K.K.). The obtained rHb(βK120C) had a concentration of approximately 100 mg L^−1^ of media. The sulfhydryl group assay of rHb(βK120C) was conducted by reaction with 4,4′-dithiodipyridine (4,4′-DTP).^35^

### Preparation of MA-HSA

The 25% HSA solution (4 mL) was diluted with PBS solution (14 mL, pH 7.4). A DMSO solution of BMH (7.5 mM, 2 mL) was then added dropwise to the HSA solution (8.4 mM, 18 mL). After stirring for 3 h at 25 °C, the reactant was subjected to a GFC column (Sephadex G25 superfine; GE Healthcare UK Ltd) to remove the unreacted cross-linker. The protein concentration was measured using a protein assay kit (Pierce 660 nm), whereas the sulfhydryl group assay of MA-HSA was conducted by reaction with 4,4′-DTP. The MA-HSA solution was stored at −80 °C.

### Preparation of rHb(βK120C)–HSA_2_

The MA-HSA and rHb(βK120C) solutions were mixed and concentrated to 6 mL ([MA-HSA] = 0.4 mM, [rHb(βK120C)] = 0.1 mM). The reactant was stirred under dark conditions for 24 h at 4 °C. An aliquot of the reaction mixture was analysed using SEC on an HPLC system (AKTA purify; GE Healthcare UK Ltd) equipped with an SEC column (Superdex 200 Increase, 5/150 GL; GE Healthcare UK Ltd) and using PBS solution (pH 7.4) as the mobile phase. The reaction mixture was purified using AEC using a Q Sepharose Fast Flow column (GE Healthcare UK Ltd). The mixture solution was diluted with the same volume of water and loaded onto the column equilibrated with PBS solution. The column was then washed using 10 mM PB (pH 7.4) solution. The rHb(βK120C)–HSA_2_ trimer was eluted with a 10 mM PB (pH 6.0) + 120 mM NaCl solution. Under this condition, the HSA dimer remained in the column. The collected solution was concentrated and subjected to GFC using a Superdex 200 pg column (GE Healthcare UK Ltd) to isolate the rHb(βK120C)–HSA_2_ trimer (yield: 50%). The total protein and Hb concentrations were measured using a protein assay kit (Pierce 660 nm; Thermo Fischer Scientific Inc.) and the extinction coefficient of cyano-metHb (*ε*_541_ = 4.4 × 10^4^ M^−1^ cm^−1^), respectively. The average [HSA]/[rHb(βK120C) unit] ratio of the product was estimated to be 2.0. The sulfhydryl group assay of MA-HSA was conducted *via* the reaction with 4,4′-DTP.

### Molecular dynamics simulations

MD simulations were performed using the Desmond application implemented in a Maestro graphical interface.^44,45^ We used the optimized potentials for liquid simulations (OPLS_2005) force field implemented in the Desmond software for all molecules in the system.^46^ We constructed a half model of rHb(βK120C) [αβ(K120C)–HSA model] because rHb(βK120C)–HSA_2_ has a *C*_2_ symmetry, and there has to be a sufficient distance between the HSAs for their interactions to be negligible. The crystal structure of native Hb (PDB ID: 2DN1)^37^ was used for the αβ(K120C) model. Lys-β120 was replaced with Cys using the PyMOL software (PyMOL Molecular Graphics System Version 2.0, Schrödinger, LLC.). For the HSA moiety, we used the crystal structure of HSA (PDB ID: 1AO6).^38^ The missing atoms of the side chains in the HSA were added to the model using the PyMOL software. The water molecules in the model were removed. The bond orders were assigned, and the hydrogen atoms were added to the models using the Protein Preparation Wizard tool^47^ in the Maestro software. The αβ(K120C) and HSA models were covalently linked using the BMH cross-linker [Cys-β120(αβ)-BMH-Cys-34(HSA)] and arranged so that sufficient distance was maintained between them (Fig. S2A, ESI†). The Cys-β120(Hb), Cys-34(HSA), and BMH moieties were minimized using the 3D builder in the Maestro software. The hydrogen networks and the protonated states were optimized to pH 7 using the Protein Preparation Wizard for which the p*K*_a_ values of the protein residues were predicted using PROPKA.^48^ The αβ(K120C)–HSA model was solvated using explicit TIP3P water^49^ with a buffering distance of 10 Å in an octahedral box. Sodium counterions were added to neutralize the charges. The model was relaxed using a default relaxation protocol implemented in the Desmond software. An isothermal–isobaric MD simulation was performed for 50 ns to maintain the 300 K temperature and 1.01325 bar pressure. The main chains (Cα and amide atoms) of the αβ(K120C) moiety were frozen during the simulation. The time step was 2 fs, and the information for analysis was printed every 100 ps. A 10 Å cut off was used for non-bonded interactions.

### Preparation of oxy and deoxy forms of the rHb(βK120C)–HSA_2_ trimer

The oxy (O_2_ complex) rHb(βK120C)–HSA_2_ trimer solution (PBS, pH 7.4, 3 μM, 3 mL) was prepared using our previously reported technique.^29^ The solution was transferred to an optical quartz cuvette (10 mm path length) with a rubber septum cap. N_2_ gas was blown into the oxy-form solution to yield the deoxy rHb(βK120C)–HSA_2_ trimer. The UV-vis absorption and CD spectra of these species were recorded at 25 °C.

### O_2_ binding parameters

The O_2_ affinity (*p*_50_; O_2_ partial pressure where Hb is half-saturated with O_2_) and Hill coefficient (*n*) were determined using an automatic recording system for the O_2_ equilibrium curve (Hemox Analyzer; TCS Scientific Corp.) at 37 °C. The oxy-form of the rHb(βK120C)–HSA_2_ trimer solution (PBS, pH 7.4, approximately 5 μM, 4 mL) was used for the measurements. The trimers in PBS solution (pH 7.4) were deoxygenated by flushing with N_2_ and oxygenated by increasing the O_2_ partial pressure.

### O_2_ complex stability

The oxy form stability of the rHb(βK120C)–HSA_2_ trimer was assessed using the first-order autoxidation rate constant (*k*_ox_) of the core Hb using our earlier described procedures.^50^

## Conflicts of interest

There are no conflicts to declare.

## Supplementary Material

CB-001-D0CB00056F-s001

## References

[cit1] Perutz M. F., Muirhead H., Cox J. M., Goaman L. C. G. (1968). Nature.

[cit2] Perutz M. F. (1970). Nature.

[cit3] Baldwin J., Chothia C. (1979). J. Mol. Biol..

[cit4] Fermi G., Perutz M. F., Shaanan B., Fourme R. (1984). J. Mol. Biol..

[cit5] Eaton W. A., Henry E. R., Hofrichter J., Mozzarelli A. (1999). Nat. Biol..

[cit6] Adachi S., Park S. Y., Tame J. R. H., Shiro Y., Shibayama N. (2003). Proc. Natl. Acad. Sci. U. S. A..

[cit7] Jones E. M., Balakrishnan G., Spiro T. G. (2012). J. Am. Chem. Soc..

[cit8] Viappiani C., Abbruzzetti S., Ronda L., Bettati S., Henry E. R., Mozzarelli A., Eaton W. A. (2014). Proc. Natl. Acad. Sci. U. S. A..

[cit9] Shibayama N., Sugiyama K., Tame J. R., Park S. Y. (2014). J. Am. Chem. Soc..

[cit10] Jones E. M., Monza E., Balakrishnan G., Blouin G. C., Mak P. J., Zhu Q. H., Kincaid J. R., Guallar V., Spiro T. G. (2014). J. Am. Chem. Soc..

[cit11] Yuan Y., Tam M. F., Simplaceanu V., Ho C. (2015). Chem. Rev..

[cit12] Shibayama N., Sato-Tomita A., Ohki M., Ichiyanagi K., Parkc S. Y. (2020). Proc. Natl. Acad. Sci. U. S. A..

[cit13] Arnone A. (1972). Nature.

[cit14] Richard V., Dodson G. G., Mauguen Y. (1993). J. Mol. Biol..

[cit15] Ho C., Russu I. M. (1987). Biochemistry.

[cit16] Fang T.-Y., Zou M., Simplaceanu V., Ho N. T., Ho C. (1999). Biochemistry.

[cit17] Mozzarelli A., Ronda L., Faggiano S., Bettati S., Bruno S. (2010). Blood Transfus..

[cit18] Meng F., Kassa T., Jana S., Wood F., Zhang X., Jia Y., D’Agnillo F., Alayash A. I. (2018). Bioconjugate Chem..

[cit19] Schultz S. C., Grady B., Cole F., Hamilton I., Burhop K. (1993). J. Lab. Clin. Med..

[cit20] Doherty D. H., Doyle M. P., Curry S. R., Vali R. J., Fattor T. J., Olson J. S., Lemon D. D. (1998). Nat. Biotechnol..

[cit21] Chatterjee R., Welty E. V., Walder R. Y., Pruitt S. L., Rogers P. H., Arnone A., Walder J. A. (1986). J. Biol. Chem..

[cit22] Nagababu E., Ramasamy S., Rifkind J. M., Jia Y., Alayash A. I. (2002). Biochemistry.

[cit23] Jahr J. S., Moallempour M., Lim J. C. (2008). Expert Opin. Biol. Ther..

[cit24] Kluger R., Foot J. S., Vandersteen A. A. (2010). Chem. Commun..

[cit25] Vandegriff K. D., Malavalli A., Wooldridge J., Lohman J., Winslow R. M. (2003). Transfusion.

[cit26] Manjula B. M., Tsai A., Upadhya R., Perumalsamy K., Smith P. K., Malavalli A., Vandegriff K., Winslow R. M., Intaglietta M., Prabhakaran M., Friedman J. M., Acharya A. S. (2003). Bioconjugate Chem..

[cit27] Li D., Hu T., Manjula B. N., Acharya S. A. (2009). Bioconjugate Chem..

[cit28] Tomita D., Kimura T., Hosaka H., Daijima Y., Haruki R., Ludwig K., Böttcher C., Komatsu T. (2013). Biomacromolecules.

[cit29] Funaki R., Kashima T., Okamoto W., Sakata S., Morita Y., Sakata M., Komatsu T. (2019). ACS Omega.

[cit30] Haruki R., Kimura T., Iwasaki H., Yamada K., Kamiyama I., Kohno M., Taguchi K., Nagao S., Maruyama T., Otagiri M., Komatsu T. (2015). Sci. Rep..

[cit31] Iwasaki H., Yokomaku K., Kureishi M., Igarashi K., Hashimoto R., Kohno M., Iwazaki M., Haruki R., Akiyama M., Asai K., Nakamura Y., Funaki R., Morita Y., Komatsu T. (2018). Artif. Cells, Nanomed., Biotechnol..

[cit32] Morita Y., Yamada T., Kureishi M., Kihira K., Komatsu T. (2018). J. Phys. Chem. B.

[cit33] Morita Y., Igarashi K., Funaki R., Komatsu T. (2019). ChemBioChem.

[cit34] Funaki R., Okamoto W., Endo C., Morita Y., Kihira K., Komatsu T. (2020). J. Mater. Chem. B.

[cit35] Grassetti D. R., Murry Jr. J. F. (1967). Arch. Biochem. Biophys..

[cit36] Cheng Y., Shen T. J., Simplaceanu V., Ho C. (2002). Biochemistry.

[cit37] Park S. Y., Yokoyama T., Shibayama N., Shiro Y., Tame J. R. (2006). J. Mol. Biol..

[cit38] Sugio S., Kashima A., Mochizuki S., Noda M., Kobayashi K. (1999). Protein Eng..

[cit39] Perutz M. F., Ladner J. E., Simon S. R., Ho C. (1974). Biochemistry.

[cit40] Aki-Jin Y., Nagai Y., Imai K. and Nagai M., in New Approaches in Biomedical Spectroscopy, ed. K. Kneipp, R. Aroca, H. Kneipp and E. Wentrup-Byrne, American Chemical Society, Washington, DC, 1st edn, 2007, ch. 19, vol. 963, pp. 297–311

[cit41] Nagai M., Nagatomo S., Nagai Y., Ohkubo K., Imai K., Kitagawa T. (2012). Biochemistry.

[cit42] The cooperativity reduction was determined using the following equation, cooperativity reduction (%) = (*n*_native Hb_ − *n*)/(*n*_native Hb_ − 1.0) × 100, where *n*_native Hb_ and *n* are Hill coefficient of native Hb and chemically modified Hb, respectively, and are measured in the same conditions. When the cooperativity is completely lost, the Hill coefficient becomes 1.0 and the cooperativity reduction reaches 100%

[cit43] Wiltrout M. E., Giovannelli J. L., Simplaceanu V., Lukin J. A., Ho N. T., Ho C. (2005). Biochemistry.

[cit44] *Desmond Molecular Dynamics System*, D. E. Shaw Research, Maestro-Desmond Interoperability Tools, Schrödinger LLC, New York, NY, 2016

[cit45] *Maestro*, Schrödinger Release 2017-3, Schrödinger LLC, New York, NY, 2017

[cit46] Banks J. L., Beard H. S., Cao Y., Cho A. E., Damm W., Farid R., Felts A. K., Halgren T. A., Mainz D. T., Maple J. R., Murphy R., Philipp D. M., Repasky M. P., Zhang L. Y., Berne B. J., Friesner R. A., Gallicchio E., Levy R. M. (2005). J. Comput. Chem..

[cit47] Schrödinger Suite 2012 *Protein Preparation Wizard*, LLC, New York, NY, 2012; *Impact version 5.8*, Schrödinger, LLC, New York, NY, 2012

[cit48] Olsson M. H. M., Søndergard C. R., Rostkowski M., Jensen J. H. (2011). J. Chem. Theory Comput..

[cit49] Jorgensen W. L., Chandrasekhar J., Madura J. D., Impey R. W., Klein M. L. (1983). J. Chem. Phys..

[cit50] Yamada K., Yokomaku K., Kureishi M., Akiyama M., Kihira K., Komatsu T. (2016). Sci. Rep..

