# Supplementary material for: Haemoglobin(βK120C)–albumin trimer as an artificial O2 carrier with sufficient haemoglobin allostery
Source: RSC Chem Biol. 2020 Jul 13;1(3):128–36. doi: 10.1039/d0cb00056f (PMC8341959; doi:10.1039/d0cb00056f)
Supplement: CB-001-D0CB00056F-s001 [file CB-001-D0CB00056F-s001.pdf]

Electronic Supplementary Material (ESI) for RSC Chemical Biology.
This journal is © The Royal Society of Chemistry 2020

Haemoglobin(βK120C)–albumin trimer as artificial O_2 carrier with sufficient haemoglobin allostery

Yoshitsugu Morita,[a] Asuka Saito,[a] Jun Yamaguchi,[a] and Teruyuki Komatsu*[a]

[a]Department of Applied Chemistry, Faculty of Science and Engineering, Chuo University, 1-13-27 Kasuga, Bunkyo-ku, Tokyo 112-8551, Japan

Corresponding author: Prof. Teruyuki Komatsu

Tel & Fax: +81-3-3817-1910, E-mail: komatsu@kc.chuo-u.ac.jp

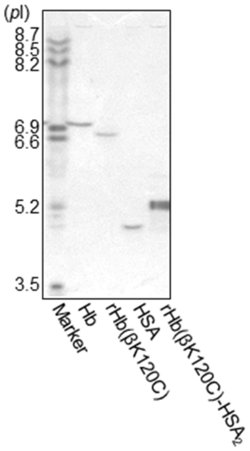

Fig. S1 Isoelectric focusing results of Hb, rHb(βK120C) and rHb(βK120C)–HSA_2.

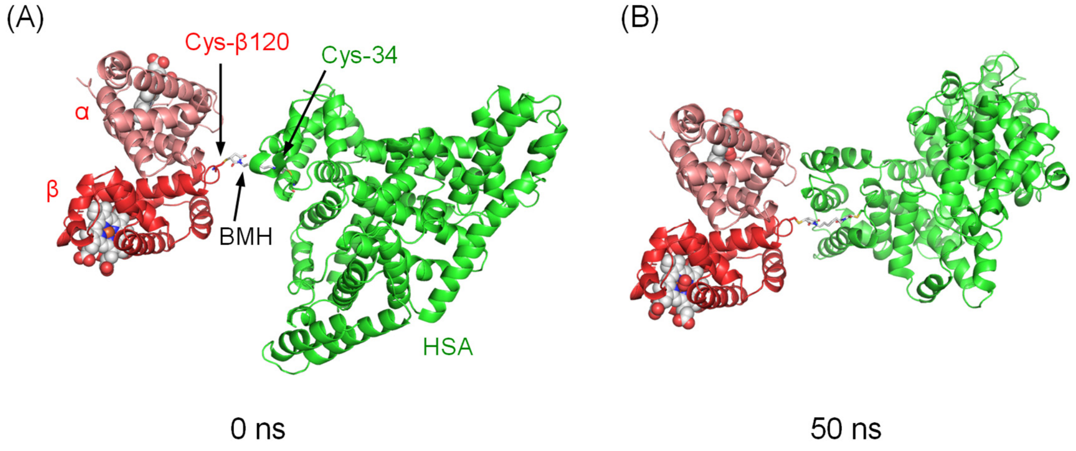

Fig. S2 (A) Initial structure of the αβ(K120C)–HSA model and (B) the MD simulated structure after 50 ns. The model was constructed based on crystal structures of Hb (PDB ID: 3DN1) and HSA (PDB ID: 1AO6).

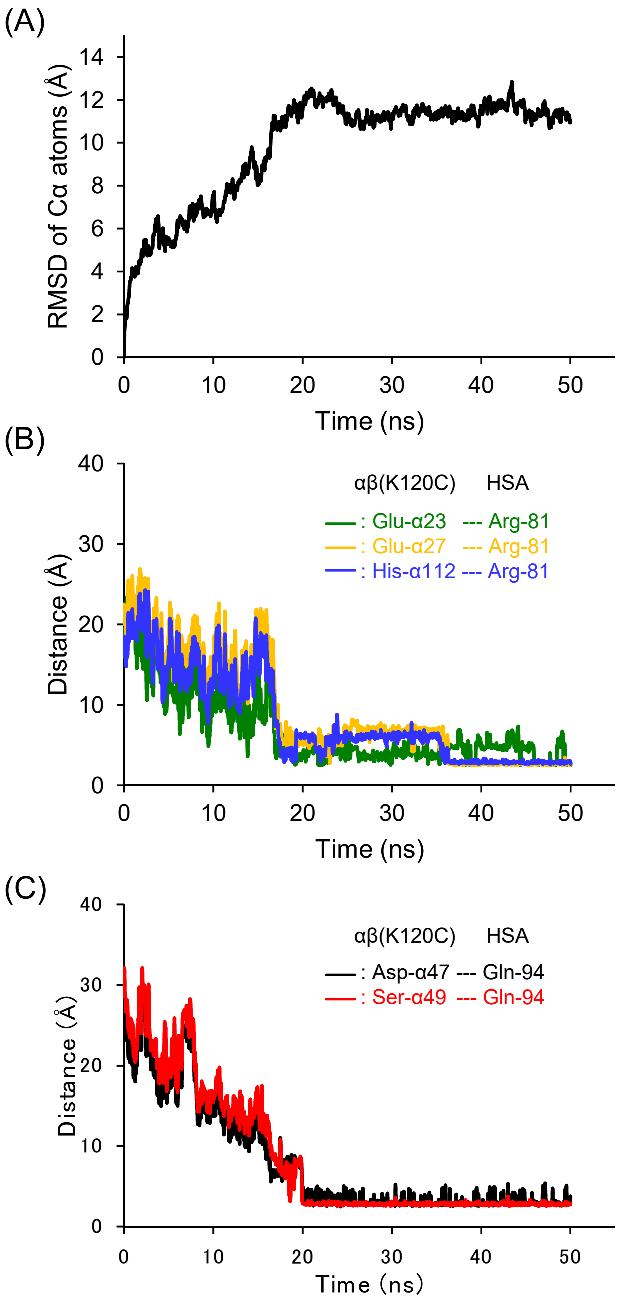

Fig. S3 (A) Time evolution of the RMSD of Ca atoms from the staring structure during 50 ns MD simulation. (B,C) The plots of distances for Glu-α23[αβ(K120C)]–Arg-81(HSA) (green), Glu-α27[αβ(K120C)]–Arg-81(HSA) (yellow), His-α112[αβ(K120C)]–Arg-81(HSA) (blue), Asp-α47[αβ(K120C)]–Gln-94(HSA) (black), and Ser-α49[αβ(K120C)]–Gln-94(HSA) (red) of the αβ(K120C)–HSA model against the simulation time.

Table S1 UV-vis absorption spectral data of the haemeproteins in PBS solution (pH 7.4) at 25 °C

Haemeprotein	λ_{max} (nm)		
	deoxy	oxy	carbonyl
Hb	430, 555	415, 541, 577	419, 540, 569
rHb(βK120C)	430, 555	414, 541 ,577	419, 538, 569
rHb(βK120C)–HSA_2	429, 553	414, 541, 577	419, 538, 569

Table S2 O_2 binding parameter for the Bohr effect on the haemeproteins in PBS solution at 37 °C

Haemeprotein	P_{50} (Torr)				
	pH 8.0	pH 7.4	pH 7.0	pH 6.5	pH 6.2
Hb	9	12	17	23	28
rHb(βK120C)	9	12	16	23	29
rHb(βK120C)–HSA_2	8	12	16	23	28
